# Germans’ awareness of refugees’ information barriers regarding health care access: a cross-sectional study

**DOI:** 10.1186/s12913-023-09226-9

**Published:** 2023-03-07

**Authors:** Saskia Schubert, Ulrike Kluge, Felix Klapprott, Tobias Ringeisen

**Affiliations:** 1 Berlin School of Economics and Law, Alt-Friedrichsfelde 60, 10315 Berlin, Germany; 2 Klinik für Psychiatrie und Psychotherapie, Charité Universitätsmedizin Berlin, Bonhoefferweg 3, 10117 Berlin, Germany; 3 Psychologische Hochschule Berlin, Am Köllnischen Park 2, 10179 Berlin, Germany

**Keywords:** Empathy, Positive intercultural contact, Refugees, Health care services, Attitudes

## Abstract

**Background:**

In light of their experiences on the refuge and upon their arrival in the receiving society, refugees may have differentiated needs regarding health care. However, negative attitudes of the members of the receiving society and a lack of information pose as barriers for refugees when trying to access health care services. In that sense, it is largely unknown, which antecedents positively affect Germans’ perception of information barriers that refugees face. Based on an extended version of the Empathy-Attitude-Action model, this study examined selected predictors of problem awareness in the form of perceived information barriers that refugees face, emphasizing the role of positive intercultural contact experiences.

**Methods:**

A sample of members of the receiving society, here: Germans (N = 910) completed a cross-sectional online survey with validated self-report measures. From the perspective of Germans, assessments covered positive intercultural contact, attitudes on refugees’ rights, the recognition of refugees’ socio-emotional support needs as a form of cognitive empathy, and the perception of refugees’ information barriers when accessing health care. We conducted structural equation modeling to examine hypothesized latent associations and specified three different models with unidirectional paths between the study variables, each allowing another direct path from intercultural contact to the variables. We determined the best model using the chi-square-difference test and tested for indirect effects along the paths through bias-corrected bootstrapping.

**Results:**

Our results show consistency with the Empathy-Attitude-Action model. We found Germans’ cognitive empathy toward refugees to be associated with more positive attitudes and a greater awareness of refugees’ information barriers. We further found more positive intercultural contact to be associated with greater cognitive empathy toward refugees and with more positive attitudes. While these contact experiences showed a slightly direct negative effect on Germans’ perception of refugees’ information barriers to accessing health care, the indirect effects via cognitive empathy and positive attitudes were positive.

**Conclusion:**

Previous positive intercultural contact may be directly and indirectly linked to greater awareness for refugees, helping Germans as the receiving community (1) to become more empathetic toward refugees, (2) to improve their attitudes toward refugees’ rights and to (3) raise consciousness for information barriers that refugees face when trying to access health care services.

In the last ten years, the number of displaced people around the world has doubled. Due to wars, violent conflicts, and persecution 82.4 million people worldwide were forced to flee and abandon their homes, friends, and families of whom 1.23 million refugees found shelter in Germany [1]. With that, the country hosted the fifth-largest number of people displaced across country borders worldwide [1]. For instance, 1,056,416 Ukrainian refugees have been registered in Germany since the war started in February 2022, with a further influx to be expected with the war still going on [2].

Such high numbers of refugees pose a challenge for the health care infrastructure of receiving countries such as Germany, as refugees face multiple stressors throughout the process of arriving and resettlement that require access to adequate health care. Aside from traumatic experiences during the flight, especially social stressors such as a lack of support in daily life, rejection or even discrimination can lead to multiple psychological issues that make it necessary to receive treatment [3–6]. In terms of a downward spiral, refugees’ health may further deteriorate if their health care needs are met insufficiently [7].

Despite the great need for adequate health care, refugees in Germany often report a lack of knowledge about how to access and use health care [8]. Healthcare for citizens in Germany is financed by the statutory health insurance scheme, ensuring the coverage of treatment. However, the medical care for refugees seeking asylum in Germany is based on the Asylum Seekers Benefits Act (in German “Asylbewerberleistungsgesetz”) [9], which determines that asylum seekers are entitled to the treatment of acute illnesses and pain, including necessary medical and dental treatment and supply of medicines or other services required for recovery from illnesses. Particularly vulnerable persons, such as pregnant women, minors, traumatized persons, or persons with disabilities, are entitled to further necessary medical care.

In case of an acute need for medical treatment, there are different procedures that give refugees who are registered as asylum seekers access to medical care, depending on the federal state laws in Germany. In six federal states, refugees can receive electronic health cards, which facilitate access to health care and offer a more extensive medical care; three other federal states are currently in the process of implementing the card. In the remaining seven federal states, the responsible municipal offices issue treatment vouchers that refugees can use to see a doctor [9].

It is the responsibility of the government and the federal state offices to ensure treatment and provide refugees with access to appropriate information on the health care system in general, on their support options in particular or where to go in order to get help [9, 10]. In everyday life, however, refugees tend to experience rather the opposite. Bureaucratic, complicated, and legally strenuous processes pose a barrier for refugees when trying to obtain adequate information about accessing and utilizing health care services [9, 10]. Negative experiences with state and/or health authorities, e.g. due to language barriers or distrust can reinforce an already existing lack of information or a reluctance to try accessing health care services at all [8, 11, 12].

If public authorities do not adequately provide refugees with relevant information, members of the host community could serve as gatekeepers^1^ and help refugees to become familiar with health care options and appropriate contacts, provided they are aware of the information barriers that refugees often face [13]. With this in mind, it is important to find out, how Germans can help to facilitate refugees’ access to health care services. This includes exploring Germans’ awareness of refugees’ health care information barriers and its antecedents, as increased awareness may provide a bridge to taking supportive action [13]

Antecedents to awareness and the intention to help may include empathy, positive attitudes, and previous positive contact experiences with people of other cultural backgrounds [14–16]. However, there is little research on the interplay of these factors in the context of refugee support in Germany [17, 18]. Existing research on refugees has focused on negative attitudes that not only cause and exacerbate refugees’ mental health issues, but may also obstruct their access to health care services [11, 19, 20]. Building on existing research, we therefore used and extended the Empathy-Attitude-Action-model (EAA-model) [14], which specifies antecedents of prosocial action toward members of the outgroup, to examine the German receiving community’s awareness of information barriers refugees may face in accessing health care. Specifically, we examined empathy, positive attitudes, and positive contact experiences as positive antecedents of awareness.

## Using the empathy-attitude-action-model to examine Germans’ empathy and awareness toward refugees

Batson and colleagues’ EAA-model assumes that empathy toward one member of an outgroup expands to empathy toward the entire outgroup, which should promote positive attitudes toward the outgroup and lead to motivation to help [14]. Thus, outgroup attitudes are suggested to serve as a mediating variable between empathy and prosocial action intentions. Empathy consists of an emotional and a cognitive component [21, 22]. Emotional empathy can be defined as an affective response that is similar to another person’s emotional state and is based on an understanding of what the person needs to feel better (Eisenberg et al., 1991; 2010) [23, 24]. Such understanding corresponds to cognitive empathy in the sense that the perspective of another person in a given situation is consciously taken [14, 25]. In the EAA-model, attitudes are defined as the overall evaluation of an outgroup and its members, whereas prosocial action can be described as the intention to help [14]. In the context of health care, prosocial action may thus refer, for example, to informational support in terms of advice on organizations or websites that might help with getting an appointment with a specialized physician or therapist.

Since its publication in 2002, the assumptions about the enhancing effect of empathy on positive attitudes toward an outgroup and resulting prosocial behaviour, as specified in the EAA-model, have been confirmed by numerous studies with children and adolescents in the context of intercultural contact, including contact between members of the receiving society and refugees; studies with adults are sparse yet also yielded confirming evidence [14, 17, 20]. For instance, previous research has shown that encouraging people to take the perspective of the outgroup (i.e. cognitive empathy) increases emotional empathy, which in turn promotes positive attitudes toward the outgroup [26]. In a study with children in Northern Ireland that looked at their interaction with Syrian refugee children, Glen and colleagues induced empathy and found that attitudes toward the outgroup predicted children’s willingness to help incoming refugee children [15]. In another study with Italian children without migration experiences [18], intergroup empathy was found to be associated with higher levels of positive attitudes toward immigrant children and with more prosocial behavioral intentions.

These findings underline that the EAA-model is suitable for researching the antecedents of intentions to help refugees. Yet, the model and its core constructs need to be redefined in order to explore host society members’ awareness of the information barriers which refugees face to accessing health care. As a starting point, Germans need to be able to take the perspective of refugees who may have suffered multiple stressful experiences during refuge, arrival and resettlement, resulting in severe emotional disturbances that require appropriate treatment [3–6]. Therefore, with regard to empathy, we specifically examined cognitive empathy in our study to assess whether Germans recognize refugees’ socio-emotional support needs as a form of perspective-taking [25]. The vast majority of receiving society members have no or at most little contact with refugees and thus most likely could not develop emotional empathy [6, 27].

Given the role of outgroup attitudes when predicting prosocial action, positive attitudes are crucial for peoples’ willingness to offer help to members of the outgroup [14, 18]. Negative attitudes against refugees and their rights, on the other hand, reduce prosocial behaviour among members of the receiving society [20]. Positive attitudes toward refugees may thus pose as a counterweight to negative attitudes, and enhance the willingness of ingroup members to provide information on how refugees can use and access health care. Acknowledging refugees’ rights would mean to grant them better access to health care services, as the recognition of a legal asylum status would grant refugees better access to state/public services such as higher education, health care, etc [9]. We therefore operationalized Germans attitudes toward refugees through their positive attitudes regarding refugees’ rights.

In addition to redefine the three core constructs, we suggest adapting the EAA-model by including awareness regarding access barriers that refugees face as a prerequisite for prosocial action. As Germans so far reported low to no contact with refugees [6], actual helping action cannot be measured in a valid way. However, prior research suggests that awareness of access barriers to health care can be seen as an important precondition to the motivation of providing informational support [13, 28]. In our context, Germans as members of the receiving society need to perceive refugees’ information barriers, when trying to use health care services to jump into action and provide information on health care access [13]. Therefore, in line with Batson et al., [14] we concentrated on Germans’ perception of refugees’ information barriers regarding health care as an antecedent of helping actions as our outcome variable.

**Fig. 1:**
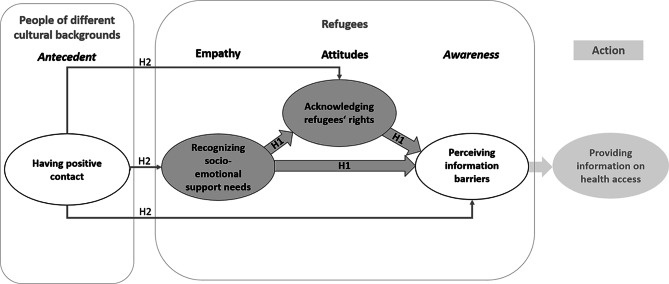
Extended EAA-model including awareness positive contact with people of different cultural background as an antecedent. *Note.* Dark grey shapes are variables from the original EAA model, white shapes are extensions of the original model, the light grey frames represent the respective outgroup, and the arrows visualize the expected positive relationships according to H1 and H2

Based on the refined EAA-model and the study findings by Yaya et al. from 2019 [13], we specified our first hypothesis: We propose that Germans’ cognitive empathy toward refugees is linked to positive attitudes toward refugees and their rights, and positively connected to the awareness of information barriers, as visualized with bold arrows in Fig. 1. Accordingly, to perceive refugees’ information barriers and be able to support them on their way to getting access to health care, Germans first would have to recognize their socio-emotional support needs and acknowledge their rights. We expected greater cognitive empathy among Germans to be linked to a greater awareness of refugees’ information barriers regarding access to health services, directly and indirectly via more positive attitudes regarding refugees’ rights (see Fig. 1, H1).

## The role of positive intercultural contact for empathy and attitude development

In the present study, we propose to extend the EAA-model by including previous positive intercultural contact experiences as a relevant antecedent in the model, as they have been found in previous studies to significantly predict empathy, attitudes, and awareness toward socio-cultural outgroups, and the intention to help these outgroups [16, 29]. However, it is not yet known whether positive contact with people of different cultural backgrounds enhances perceptions of information barriers, and whether this effect is direct or mediated by empathy or outgroup attitudes, as suggested by the EAA-model. Previous studies primarily examined the effects of cross-ethnic friendships and empathy on attitudes toward culturally or ethnically diverse groups and on participants’ motivation to engage in helping behaviour. For instance, in a study with children from different ethnic backgrounds, Aboud and colleagues found that intergroup contact was associated with positive attitudes toward children with a different ethnic background [29]. Studies conducted among children with and without a migration background in Germany and Italy found similar results [18, 30]. In addition, and in support of the EAA-model, Vezzali et al. found that positive attitudes could lead to an increase of motivation to act helpfully [18]. Johnston and Glasfords study with adults of different ethnic backgrounds confirmed associations between empathy and outgroup prosocial intentions mediated by attitudes [31].

However, research on contact between refugees and members of the receiving society is sparse [18], likely because the vast majority of adults in the receiving society report having little or no contact with refugees [27]. Therefore, we decided to assess previous positive intercultural contact experiences with people from different cultural backgrounds in general, as positive experiences with one socio-cultural outgroup are positively linked to empathy, supportive attitudes, awareness, and intentions to help other socio-cultural outgroups [6, 18].

Extending the EAA, we therefore propose in our second hypothesis that prior positive contact with people of different cultural backgrounds serves as a relevant antecedent, which shows positive and direct associations with cognitive empathy, positive attitudes and awareness of refugees’ information barriers (see Fig. 1, H2). Therefore, previous positive intercultural contact should help Germans to perceive greater socio-emotional support needs of refugees to a greater extent, improve their attitudes toward refugees’ rights, and enable them to develop greater awareness of refugees’ information barriers when accessing health services. Further, we expect indirect associations between having positive contact with people of different cultural backgrounds and with the awareness of information barriers via cognitive empathy and positive attitudes. Figure 1 shows the extended EAA-Model. Empathy, attitudes, and prosocial action as core constructs of the original EAA-model are shown in grey, with action masked out because we did not examine this variable in the present study. Instead, we included awareness of information barriers, and further, intercultural contact as an antecedent, which are shown in white. The light grey frames illustrate the outgroup context of each variable.

## Method

### Sample

The sample consisted of 910 Germans (Mage = 48.40, SD = 14.79) of whom 460 identified themselves as female (50.5%) and 450 as male (49.5%). All participants met three criteria to be included in the study: They reported to (1) have German citizenship, (2) be born in Germany, and (3) their parents to be born in Germany as well.^2^ We focused on Germans without migration experience because we wanted to find out how people whose intercultural contact experiences had predominantly been collected outside the family context empathize with refugees and perceive their access to health care.

All participants were aged 18 or older. Concerning education, three participants did not complete schooling; 148 completed secondary school qualification (8th grade), 379 completed the secondary school certificate (10th grade), 188 completed A-levels with a higher education entrance qualification, and 192 completed a degree at university or college [6].

### Procedures

After approval by the Institutional Review Board and all relevant administrative units, an internet survey company was used to conduct an online survey. To generate an approximately representative adult sample in terms of age, gender, education, and place of residence in accordance with the German Bureau of Statistics, the survey company drew a random sample of 2086 from a panel of more than half a million Germans, who had previously provided consent to be contacted for survey purposes. This sample was invited to participate in the survey via a link sent to them by the survey company (response rate = 44%). Before the survey began, participants received detailed written instructions on how to complete the questionnaires. They were explicitly informed that participation in the study was voluntary and that all their answers would be treated confidentially, and that participants were not obliged to answer a question if they felt uncomfortable doing so. When starting the survey, participants provided written informed consent. Respondents had to answer three filter questions with ‘yes’ to be included in the survey to ensure that only Germans participated (do you have German citizenship: yes/no; were you born in Germany: yes/no), were both your parents born in Germany: yes/no). Completing the questionnaire took less than 20 minutes [6].

### Measures

From the perspective of the Germans as the receiving society, we used established self-report instruments validated on German-speaking samples to assess positive contact with people of different cultural backgrounds, recognition of refugees’ socio-emotional support needs, positive attitudes toward refugees’ rights, and the perception of refugees’ information barriers when accessing health care. Except for positive intercultural contact, item wording and/or instructions were adapted to assess them with reference to refugees who had arrived in Germany in previous years, for instance by changing the reference group from “migrants” to “refugees”) [6, 8]. After adjustment the wording and/or the introductory statements of the respective measures, we piloted the changes. Six Germans varying in terms of age, gender and educational background checked the adjusted instructions and/or respective items for clarity and understandability. As all items were approved and no potential for further modification was identified, we proceeded with the study.

#### Antecedent

We used a subscale from the German adaption of the Intercultural Sensitivity Scale with the title “Enjoyment of intercultural interactions” to examine *positive intercultural contact* as an antecedent of empathy, attitudes, and awareness [32, 33]. The subscale includes four statements that capture attitudes and affective responses to intercultural situations, accompanied by a four-point Likert scale (1=“strongly disagree” to 4=“strongly agree”. One item example states “I gladly socialize with people from different cultures.” Factor loadings in the original study ranged from 0.52 to 0.83; the corresponding Cronbach’s Alpha was 0.87 [33]. In the current study, factor loadings ranged from 0.86 to 0.89 and Cronbach’s Alpha was 0.91. We chose to measure positive contact with people from different cultural backgrounds in general rather than with refugees in particular. There were two reasons for this decision. First, refugees are a very heterogeneous group in terms of ethnicity, cultural background, and country of origin that members of the receiving society can hardly distinguish from other migrants in everyday interactions [34]. Second, the vast majority of Germans have no or at most little contact with refugees. Under these conditions, positive contact with refugees cannot be validly captured [6, 27].

#### Empathy

Recognizing socio-emotional support needs as an operationalization of cognitive empathy was assessed with a subscale consisting of three items of the Berlin Social Support Scale (BSSS) [35]. The original scale includes four items. However, one item was removed that reflected the need for nonspecific support and had a low factor loading of 0.26 in the original validation study. The wording was slightly adjusted to reflect the perspective of Germans on refugees. An item example is “When refugees are down, they need someone who boosts their spirits.” The items were provided with a Likert scale ranging from 1 (“strongly disagree”) to 4 (“strongly agree”). In the original study, factor loadings for the three-item scale, excluding the aforementioned low factor loading item ranged from 0.46 to 0.54; Cronbach’s Alpha was 0.73. In our study, the loadings were between 0.73 and 0.92. Alpha was 0.89.

#### Attitudes

Positive attitudes toward refugees’ rights, which would grant refugees better access to public services such as health care, were assessed using a three-item subscale of Eurobarometer 53 validated by Manzoni [36, 37]. The items were provided with a Likert scale ranging from 1 (“strongly disagree”) to 4 (“strongly agree”). An item example is “Naturalization should be eased for refugees with residence.” In our study, the loadings ranged from 0.66 to 0.82. Cronbach’s Alpha was 0.78.

#### Awareness

Awareness of information barriers that refugees face in accessing health care was measured using a four-item scale developed by Maier et al. [8] Initially, this scale was develop to assess the perceived information barriers that migrants may encounter when accessing the health care system in Germany. The wording was slightly adjusted to reflect the awareness of Germans for the information barriers that refugees may face. The instruction was “Please estimate the extent to which refugees are familiar with using health services in Germany.” The items were provided with a Likert scale ranging from 1 (“not at all”) to 4 (“a great deal”). An example of an item is “Refugees don’t receive the information they need.” Factor loadings originally ranged from 0.63 to 0.76 and Alpha was 0.88. In our study, the loadings ranged from 0.82 to 0.89. Cronbach’s Alpha was 0.92.

### Statistical analysis

We used Mplus version 8.00 to examine the hypothesized latent associations between the study variables using structural equation modeling (SEM) [38]. Age and gender (1 = male, 2 = female), the level of education, and economical status in terms of monthly income were included as covariates in the model.

In a first step, we conducted a multifactorial confirmatory factor analysis (CFA) to evaluate the measurement model and determine the latent correlations among the study variables (Model 1). We then specified different SEMs with unidirectional paths between the study variables, reflecting the assumptions of the modified EAA-model on the relationships among cognitive empathy, attitudes, awareness of information barriers, and the antecedent of having positive intercultural contact. To examine whether positive intercultural contact displays direct relations with empathy, attitudes, and awareness, we computed three different models and gradually increased the number of direct paths from contact to the remaining study variables. Model 1 as the baseline model included direct effects from intercultural contact on cognitive empathy, from empathy on positive attitudes, and from attitudes to awareness of information barriers. In Model 2, we added a direct path from contact on attitudes. In Model 3, we included an additional path from contact on perceived information barriers. Model 1 was tested against Model 2, and Model 2 against Model 3, using the χ2-difference test [39]. If the test yields a significant result, this indicates that the less restrictive model, which considers an additional path, fits the data better. In the final step, we tested for indirect effects using bootstrapped confidence intervals (boot = 2000). As bootstrapped confidence intervals are not available with MLR estimation, we used the confidence intervals from an analogous model with ML estimation.

The Satorra-Bentler method for model estimations was used for all analyses. This approach yields maximum likelihood parameter (MLR) estimates and a mean-adjusted χ2 value that is robust to violations of normality of item distributions [38]. Model fit was estimated using primary fit indices as recommended by Hu and Bentler: The Chi-Square Test of Model Fit (χ2), the Root Mean Square Error of Approximation (RMSEA) including 90% confidence intervals, the Comparative Fit Index (CFI), the Tucker-Lewis Index (TLI), and the Standardized Root Mean Square Residuals (SRMR) [40]. For the CFI and the TLI, a value close to 1 represents excellent model fit, and a value > 0.95/0.90 a good/acceptable model fit. For the SRMR and RMSEA, a value close to 0 denotes a perfect model fit, while values ≤ 0.06/0.08 are good/acceptable [6, 40].

## Results

### Preliminary analysis

Multifactorial CFA was performed to evaluate the measurement model and to determine latent correlations among study variables. Indices for the original model showed a good fit (χ² = 196.014, df = 95, p < .0001, CFI = 0.986, TLI = 0.980, RMSEA = 0.034, CIs (0.027-0.041), SRMR = 0.023). Factor loadings for having positive intercultural contact ranged from 0.86 to 0.90, for recognizing socio-emotional support needs from 0.75 to 0.93, for acknowledging refugees’ rights from 0.66 to 0.83, and for perceiving information barriers of refugees from 0.83 to 0.89. When latent correlations were screened, the hypothesized patterns were largely confirmed (see Table 1). In addition, the correlations between the latent constructs and the demographic covariates of age, gender, education level, and economic status were examined. Educational level was positively associated with all four variables, meaning that higher education was related to more positive intercultural contact (ß = 0.22, p < .001), better recognition of socio-emotional support needs (ß = 0.18, p < .001), greater acknowledgement of refugees’ rights (ß = 0.23, p < .001), and to greater perceived information barriers (ß = 0.15; p < .001). Older participants reported more positive contact (ß = 0.08; p = .03), better recognition of socio-emotional support needs (ß = 0.12; p < .001), yet a lower perception of information barriers for refugees regarding access to health services (ß = − 0.10; p = .002). Gender was only associated with awareness of information barriers, with men perceiving more barriers (ß = − 0.10; p = .002), while economic status showed no associations with the model variables.

**Table 1:** Latent correlations of the study variables

Constructs	M	SD	1	2	3	4
1. Positive intercultural contact			-	0.64**	0.66**	0.39**
2. Recognition of socio-emotional support needs of refugees				-	0.72**	0.58**
3. Positive attitudes toward refugees’ rights					-	0.62**
4. Awareness of refugees’ information barriers						-

N = 910; **p ≤ .01.

### Relations among positive contact, empathy, positive attitudes, and awareness of barriers

**Fig. 2:**
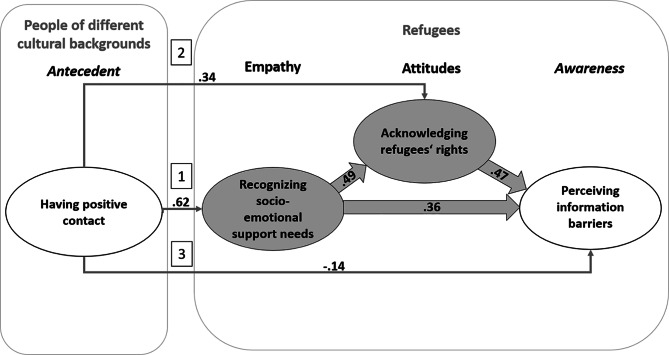
Final model with latent relations between the study variables. Note: Numbers in squares mark the added paths in model 1, model 2, and model 3

In a second step, SEMs with unidirectional paths were specified to control for variance overlap between the measures and to determine the unilateral latent relations between the variables. To determine the patterns of association between positive intercultural contact and the other study variables, three versions of the SEM were computed and compared. Apart from the unidirectional pathways between cognitive empathy, positive attitudes, and awareness of refugees’ information barriers, Model 1 included a direct path from positive intercultural contact on cognitive empathy (all paths are marked “1”, as shown in Fig. 2). For Models 2 and 3, we added direct pathways from positive intercultural contact to attitudes, and to awareness of information barriers, respectively (marked with “2” in Model 2, and with “3” in Model 3; see Fig. 2).

For Model 1, fit indices again reflected good fit (Hu & Bentler, 2004) (χ2 = 260.676*; df = 97; CFI = 0.977; TLI = 0.969; RMSEA = 0.043; SRMR = 0.036) [40]. Results indicate that more positive intercultural contact was related to greater recognition of refugees’ socio-emotional support needs (β = 0.63, p < .001), which in turn was related to more positive attitudes toward refugees’ rights (β = 0.72, p < .001), and greater perceived information barriers (β = 0.31, p < .001). More positive attitudes toward refugees’ rights were also associated with perceiving greater information barriers (β = 0.41, p < .001). Adding the pathway of positive intercultural contact to attitudes in Model 2 improved the model fit (χ2 = 205.159*; df = 96; CFI = 0.985; TLI = 0.979; RMSEA = 0.035; SRMR = 0.028; see Table 2 for model comparisons) indicating that direct enhancing effects of positive intercultural contact on cognitive empathy and positive attitudes toward refugees’ rights can be supported. Model 3 showed the best model fit (χ2 = 196.363*; df = 95; CFI = 0.986; TLI = 0.981; RMSEA = 0.034; SRMR = 0.024; for model comparisons, see Table 2) indicating that having positive contact with people of different cultural background serves as a direct positive antecedent of Germans’ cognitive empathy, positive attitudes toward refugees’ rights, and awareness of information barriers (see Fig. 2).

In terms of covariates, we found gender (ß = − 0.09, p = .002) to be negatively associated with awareness of information barriers, implying that men perceived fewer information barriers than women did. With increasing age, participants also expressed a smaller awareness of information barriers (ß = − 0.17, p < .001). However, age (ß = 0.09, p = .002; ß = 0.12, p < .001) and education level (ß = 0.07, p = .03; ß = 0.24, p < .001) showed positive associations with cognitive empathy and positive intercultural contact. Economic status was again unrelated to the study variables.

**Table 2:** Results of the χ2-Difference-Test

Comparison	Scaled χ2	df	p-value
Model 1 and 2	43,78644999	1	0.000
Model 2 and 3	9,701371573	1	0.002

### Indirect effects

Testing for indirect effects using bias-corrected bootstrapping revealed the following significant paths: having positive intercultural contact had an indirect reinforcing effect on awareness of refugees’ information barriers via recognizing their socio-emotional support needs (b = 0.22, p < .001, CIlower = 0.12 CIupper = 0.32, SE = 0.04, CR = 5.796), via positive attitudes toward refugees’ rights (b = 0.16, p < .001, CIlower = 0.09, CIupper = 0.24, SE = 0.03, CR = 5.251), and via the two aforementioned variables (b = 0.14, p < .001, CIlower = 0.09, CIupper = 0.22, SE = 0.03, CR = 5.430).

## Discussion

The results of our study on Germans’ perception of information barriers of refugees regarding health services are consistent with assumptions of the EAA-model and previous research [13, 14]. We found that Germans’ recognition of refugees’ emotional support needs is positively associated with the perception of refugees’ information barriers, both directly and indirectly through acknowledging refugees’ rights (e.g., regarding residence). These findings confirm our first hypothesis, which states that Germans’ cognitive empathy toward refugees is associated with more positive attitudes and a greater awareness of refugees’ difficulties in accessing health care. Our second hypothesis was partly confirmed. We found that having more positive contact with people from different cultural backgrounds was associated with greater cognitive empathy toward refugees and with more positive attitudes toward refugees. However, having positive contact had a slightly direct negative effect on the perception of refugees’ information barriers to accessing health care, although indirect effects - via recognition of refugees’ emotional support needs and acknowledging their rights– were positive.

The positive associations between positive contact, positive attitudes, and awareness of information barriers corroborate previous research [13, 15, 29–31], demonstrating that prior positive experiences with people of socio-culturally diverse backgrounds improve empathy, attitudes, and awareness toward other socio-culturally outgroups and/or other ethnic outgroups. While previous research mainly concentrated on children and adolescents [17, 20], our study extended the empirical evidence to adults.

The negative direct effect of positive contact on perceiving refugees’ information barriers occurred contrary to our expectations, especially considering its positive indirect effects via empathy and positive attitudes. This pattern implies that Germans who report having more positive contact with culturally diverse people perceive fewer difficulties in refugees’ access to information about health services in Germany, when controlling for cognitive empathy and positive attitudes toward refugees. At first glance, these findings seem to contradict previous study results that found positive intercultural contact to directly increase the motivation to act in a helpful manner [18, 31]. On second glance, however, the underlying processes may be more complex and require consideration of the interplay of all key constructs at once. Hence, having positive contact alone may not be sufficient, but must fall on fertile ground in terms of an interaction between personal characteristics and specific environmental features in order to enhance Germans’ awareness for information barriers that refugees face. The formation of empathy, and the formation of a positive attitude toward refugees, may constitute a prerequisite to translate positive intercultural contact into an increased sensitivity to refugees’ difficulties in accessing information regarding health services. Following this line of reasoning, having positive contact with culturally diverse people may increase Germans’ awareness of refugees’ information barriers if Germans recognize refugees’ emotional support needs and/or if they are more willing to acknowledge refugees’ rights (e.g., residence rights or the right to access full health care). Alternative interpretations could be that Germans who have had positive contacts in intercultural situations do not see the barriers in access to information, but in other areas they have experienced as a result of their contact experiences [12, 20]. They might also overestimate the information refugees already receive from caregivers or institutions, which may be overly optimistic, compared with the reality many refugees face regarding health services in Germany. Overall, our study provides new empirical evidence for the validity of the EAA-model for the intercultural context of Germans thinking about the target group of refugees by including and differentiating the effects of positive contact with people from a different cultural background and focusing on problem awareness as a prerequisite for prosocial actions.

### Strengths and limitations

This study has several strengths. With its focus on Germany as a receiving society and Germans’ perception of refugees’ information barriers to health services, it contributes to international research on empathy and prosocial action in the important intercultural context of flight. To our knowledge, it is the first study to examine the relationships between positive contact in intercultural settings, empathy, positive attitudes toward refugees, and awareness of their information barriers with a sample of German adults using structural equation modeling at a latent level. The number of studies examining the assumptions of the EAA-model in the context of refugees and members of the receiving society is small and research on awareness in the process of empathy and helping actions has been scarce [13, 15]. Furthermore, research to date has mostly focused on children or adolescents as the target group [15, 17, 20]. Our study therefore extends the limited empirical evidence that the assumptions of the EAA-model may also apply to adults.

However, some methodological limitations need to be considered. In this study, we could not examine causality between the study variables, due to the cross-sectional design of the study. Additionally, one could argue that the study’s reliance on self-reports limits its power. Rather than revealing actual differences, participants may have differed in their ability and willingness to report intensity levels on the study variables [41]. However, we were particularly interested in participants’ views because intercultural experiences, perceived needs, and attitudes are essentially subjective in nature. We therefore decided to focus on validated, well-established self-assessment instruments in the present study [6].

Refugees represent a very heterogeneous group in terms of their socio-cultural background. For future studies, we therefore recommend to survey empathy, attitudes and awareness as core constructs in relation to specific refugee groups in order to identify possible differences. Such differentiation might help to understand, why members of the receiving societies report little contact with refugees, and thus low awareness for refugees’ access barrier.

Because the focus of the present study is on the interplay of empathy and attitudes with the antecedent of positive contact on the one hand and problem awareness as a prerequisite for prosocial actions on the other, we decided not to examine prosocial action to ensure clarity of the study design. However, it is desirable to examine the entire process in future research and to test the effect of positive intercultural contact on prosocial action as well.

### Future directions and conclusion

Since positive contact with people from different cultural backgrounds is related to greater cognitive empathy and more positive attitudes toward refugees among Germans, this important predictor should be further researched and developed in theory and practice. The role of empathy and positive attitudes as mediators between positive contact and the awareness of information barriers of refugees suggests that these predictors need to be addressed simultaneously to improve refugees’ access to health services. Future research should therefore examine the mechanisms of action in more detail to explore the opportunities that positive contact in intercultural settings can provide to promote attitude change toward refugees and raise awareness of obstacles that outgroups are facing when trying to participate in society. As our study has shown, the interplay between environmental factors such as contact experiences and person-centered factors such as cognitive empathy is complex and deserves further exploration.

Legislation on health care for refugees in Germany is complex and extensive as it varies depending on the legal status of the refugee and differs depending on federal state laws and assumed urgency of the treatments. Relevant information, e.g. about what steps are necessary to obtain treatment or which treatments are covered in the area under the Asylum seekers benefit act often do not reach refugees sufficiently [8, 10, 42, 43]. However, good health is an important factor for social inclusion and thus for the German society as a whole [44]. German laypeople could be important supporters if they become aware of refugees emotional and informational struggles in the process of getting health care. By educating Germans about the current legal situation regarding refugees’ access to health services and the barriers refugees face when trying to get access, Germans may recognize the refugees’ needs regarding emotional support in the form of consolidation and they may be more willing to acknowledge refugees’ rights, for example, regarding their residency and accompanied health care. Media outlets such as public service broadcasting could play an important role in the education process by bringing facts about refugees’ access to health care and related struggles to a wider audience [14, 45]. Moderated social media talks about the topic, including experts and, more importantly, refugees that are affected by the lack of information on health care services could reach even more Germans. This form of campaigns could foster Germans’ empathy, more positive attitudes toward refugees and raise awareness for their access struggles [14]. Awareness of the problem and subsequent actions by general members of the receiving community (e.g. by giving a refugee information on necessary documents or addressing the issue politically) could help make health care information more accessible to refugees and would be a critical step toward a healthier society that recognizes the needs of all who live in it.

However, German lay people can only be a supporting factor but cannot replace a fast and easily accessible German health care system. The structural and legal barriers refugees face in accessing health services need to be further explored and the resulting implications put into practice. We recommend that future research directly assess the views of health care practitioners and employees from relevant administrative institutions (federal offices, immigrant authorities etc.) regarding the relationships between empathy, attitudes, and prior intercultural contact experiences. Health care practitioners within the relevant institutions may reflect societal perceptions and attitudes toward refugees on the one hand, but on the other hand may be very different because of the underlying ethics of their field to support people in need [11, 12]. Knowledge about their perspective on refugees and potential antecedents can give us clues on how to raise awareness and promote a change of attitudes toward displaced people from within the institutions to give refugees better access to the information they need in order to heal.

## Data Availability

The dataset generated and analysed during the current study are available in the zenodo repository, 10.5281/zenodo.7360136 [46]. An exemplary code, which was used to conduct structural equation modeling (SEM) in MPlus is available in the zenodo repository, 10.5281/zenodo.7360341 [47].

## Footnotes

1 Civil society cannot take over the state’s tasks, but it can supplement and bridge them in emergency and crisis situations (see discussion).

2 In addition, we asked participants to complete a validated four-item-self-report measure of social identification with being German [48]. The measure reflected a strong identification of the respondents with being German (M = 3.15, SD = 0.77).

## Abbreviations

BSSS: Berlin Social Support Scales
CFA: Confirmatory Factor Analysis
CFI: Comparative Fit Index
EAA: Empathy-Attitude-Action
MLR: Maximum Likelihood Parameter
SD: Standard Deviation
SEM: Structural Equation Modeling
SRMR: Standardized Root Mean Square Residuals
RMSEA: Root Mean Square Error of Approximation
TLI: Tucker-Lewis Index
UN: United Nations
UNHCR: United Nations High Commissioner for Refugees

## References

[1] United Nations High Commissioner for Refugees (UNHCR). Global Trends – Forced Displacement in 2021. In: UNHCR. 2021. https://www.unhcr.org/publications/brochures/62a9d1494/global-trends-report-2021.html. Accessed 5 Sep 2022

[2] Mehr als 967. 000 Menschen sind aus dem Krieg in der Ukraine nach Deutschland geflüchtet [Internet]. Bundesministerium des Innern und für Heimat. 2022 [cited 2022 Aug 23]. Available from: https://www.bmi.bund.de/SharedDocs/pressemitteilungen/DE/2022/08/ukraine_gefluechtete.html

[3] Kluge U, Aichberger MC, Heinz E, Udeogu-Gözalan U, Abdel-Fatah D (2020). Rassismus und psychische Gesundheit. Nervenarzt.

[4] Pascoe EA, Smart Richman L (2009). Perceived discrimination and health: a meta-analytic review. Psychol Bull.

[5] Ringeisen T, Mahat-Shamir M, Ben-Ezra M, Hamama-Raz Y, Schubert S, Genkova P, Riecken A (2020). Krank durch beidseitige Fremdheitserfahrung? Zur Rolle von Stressoren und Einstellungen für die Gesundheit von Einheimischen und Geflüchteten. Handbuch Migration und Erfolg.

[6] Schubert S, Mahat-Shamir M, Hamama-Raz Y, Ringeisen T (2022). Perceiving refugees as threats may backfire on one’s health: relations with intercultural antecedents and psychological distress among Germans. Curr Psychol.

[7] Nesterko Y, Jäckle D, Friedrich M, Holzapfel L, Glaesmer H (2020). Factors predicting symptoms of somatization, depression, anxiety, post-traumatic stress disorder, self-rated mental and physical health among recently arrived refugees in Germany. Confl Health.

[8] Maier I, Kriston L, Härter M, Hölzel L, Bermejo I (2015). Psychometrische Überprüfung eines Fragebogens zur Erfassung der Barrieren der Inanspruchnahme von Gesundheitsleistungen durch Personen mit Migrationshintergrund. Gesundheitswesen.

[9] Fritz A (2018). Gesundheit für Flüchtlinge: Eine unbestimmte, unübersichtliche und umstrittene Gesundheitsversorgung in Deutschland. Z für medizinische Ethik.

[10] Ärztepräsident kritisiert Organisation bei Flüchtlingsversorgung [Internet]. Tagesschau Newsticker. 2022 [cited 2022 Nov 28]. Available from: https://www.tagesschau.de/newsticker/liveblog-ukraine-dienstag-117.html#Fluechtlingsversorgung

[11] Penka S, Faißt H, Vardar A, Borde T, Mösko MO, Dingoyan D, Schulz H, Koch U, Kluge U (2015). Heinz A. Der stand der interkulturellen Öffnung in der psychosozialen Versorgung- Ergebnisse einer Studie in einem innerstädtischen Berliner Bezirk. Psychother Psychosom Med Psychol.

[12] Sandhu S, Bjerre NV, Dauvrin M, Dias S, Gaddini A, Greacen T, Ioannidis E, Kluge U, Jensen NK, Lamkaddem M, Puigpinós i Riera R, Kósa Z, Wihlman U, Stankunas M, Straßmayr C, Wahlbeck K, Welbel M, Priebe S (2013). Experiences with treating immigrants: a qualitative study in mental health services across 16 european countries. Soc Psychiatry Psychiatr Epidemiol.

[13] Yaya S, Okonofua F, Ntoimo L (2019). Men’s perception of barriers to women’s use and access of skilled pregnancy care in rural Nigeria: a qualitative study. Reprod Health.

[14] Batson C, Chang J, Orr R, Rowland J (2002). Empathy, Attitudes, and action: can feeling for a Member of a Stigmatized Group Motivate one to help the Group?. Pers Soc Psychol Bull.

[15] Glen C, Taylor L, Dautel J. Promoting prosocial behavior toward refugees: exploring the Empathy-Attitude-action model in middle childhood. In: Balvin N, Christie D, editors. Children and peace. Peace psychology Book Series. Wiesbaden: Springer; 2019. 10.1007/978-3-030-22176-8_5

[16] van Assche J (2019). Climates, and Intergroup Relations: a person × Context Approach. Physiol Belgica.

[17] Gönültaş S, Mulvey KL (2019). Social-developmental perspective on intergroup attitudes toward immigrants and refugees in childhood and adolescence: a roadmap from theory to practice for an inclusive society. Hum Dev.

[18] Vezzali L, Hewstone M, Capozza D, Trifiletti E, Di Bernardo GA (2016). Improving intergroup relations with extended contact among young children: mediation by intergroup empathy and moderation by direct intergroup contact: extended contact in young children. J Community Appl Soc Psychol.

[19] Karpenstein J, Nordheim F. Die Situation (unbegleiteter) minderjähriger und junger volljähriger Geflüchteter in Deutschland. In: Auswertung der Online-Umfrage 2019. Berlin: Bundesfachverband unbegleitete minderjährige Flüchtlinge e.V. 2019. https://b-umf.de/src/wp-content/uploads/2019/12/bumfumfrage2019_web_v03.pdf. Accessed 13 April 2021.

[20] Sims RN, Killen M, Ringeisen T, Genkova P, Leong F (2020). Antecedents and Consequences of Intergroup Attitudes: adopting a cross-cultural and intercultural perspective. Handbuch stress und Kultur.

[21] Davis MH (1994). Empathy: a social psychological approach.

[22] Duan C, Hill CE (1996). The current state of empathy research. J Couns Psychol.

[23] Eisenberg N, Fabes RA, Clark MS (1991). Prosocial behavior and empathy: a multimethod developmental perspective. Prosocial behaviour.

[24] Eisenberg N, Eggum ND, Di Giunta L (2010). Empathy-related responding: Associations with Prosocial Behavior, Aggression, and Intergroup Relations. Soc Issues Policy Rev.

[25] Eisenberg N, Fabes RA (1990). Empathy: conceptualization, measurement, and relation to prosocial behavior. Motiv Emot.

[26] Pagotto L, Voci A, Maculan V (2010). The effectiveness of intergroup contact at work: mediators and moderators of hospital workers’ prejudice toward immigrants. J Community Appl Soc Psychol.

[27] Kessler T, Fritsche I. Toleranz und Diskriminierung zwischen sozialen Gruppen. Sozialpsychologie. Basiswissen Psychologie. Wiesbaden:Springer, 2018.

[28] Biffl G. The promotion of employment and earning opportunity of women in Europe through gender mainstreaming. With special emphasis on Austria, WIFO Working Papers, No. 319, Austrian institute of economic research (WIFO), Vienna: 2008.

[29] Aboud F, Mendelson M, Purdy K (2003). Cross-race peer relations and friendship quality. Int J Behav Dev.

[30] Feddes A, Noack P (2009). Rutland. Direct and Extended Friendship Effects on Minority and Majority Children’s interethnic attitudes: a longitudinal study. Child Dev.

[31] Johnston BM, Glasford DE (2018). Intergroup contact and helping: how quality contact and empathy shape outgroup helping. Group Process Intergroup Relat.

[32] Fritz W, Graf A, Hentze J, Möllenberg A, Chen GM (2005). An examination of Chen and Starosta’s Model of Intercultural Sensitivity in Germany and United States. J Intercult Commun Res.

[33] Chen GM, Starosta WJ (2000). The development and validation of the intercultural communication sensitivity scale. Hum Commun Res.

[34] Brücker H, Hauptmann A, Sirries S. (2017). Arbeitsmarktintegration von Geflüchteten in Deutschland: Der Stand zum Jahresbeginn 2017. In: Aktuelle Berichte. 2017. https://doku.iab.de/aktuell/2017/aktueller_bericht_1704.pdf. Accessed 28 Nov 2022.

[35] Schwarzer R, Schulz U. Berlin Social Support Scales (BSSS). In: Measurement Instrument Database for the Social Science. 2013. https://www.midss.org/sites/default/files/berlin_social_support_scales_english_items_by_scale.pdf. Accessed 20 April 2021.

[36] European commission. Eurobarometer – Die öffentliche Meinung der Europäischen Union. In: Bericht Nr. 53. Generaldirektion Bildung und Kultur. Brüssel. 2000. https://ec.europa.eu/commfrontoffice/publicopinion/archives/eb/eb53/eb53_de.pdf. Accessed 28 Nov 2022.

[37] Manzoni P. Monitoring über Fremdenfeindlichkeit, rechtsextreme Orientierungen und Gewaltbereitschaft in der Schweiz. In: Machbarkeitsstudie. Bern: Fachstelle für Rassismusbekämpfung. 2007. https://www.edi.admin.ch/dam/edi/de/dokumente/FRB/Neue%20Website%20FRB/Monitoring%20und%20Berichterstattung/Vorstudien%20und%20Beitr%C3%A4ge/machbarkeitsstudie_monitoring_2007.pdf.download.pdf/Machbarkeitsstudie%20Monitoring%202007.pdf. 28 Nov 2022.

[38] Muthén LK, Muthén BO. (1998–2012). Mplus User’s Guide (7th ed.). Muthén & Muthén.

[39] Yuan KH, Bentler PM. (2004). On chi-square difference and z‐tests in mean and covariance structure analysis when the base model is misspecified. Educ Psychol Meas. 2004; doi:10.1177/0013164404264853

[40] Hu LT, Bentler PM (1999). Cutoff criteria for fit indexes in covariance structural analysis: conventional criteria versus new alternatives. Struct Equ Modeling.

[41] Bryant FB, Yarnold PR, Grimm LG (1996). Toward a measurement model of the Affect Intensity measure: a three-factor structure. J Res Pers.

[42] KBV-VV spricht sich für einen klaren Leistungsanspruch für ukrainische Flüchtlinge aus. [Internet]. Kassenärztliche Bundesverwaltung. 2022 [cited 2022 Nov 28]. Available from: https://www.kbv.de/html/2022_57220.php

[43] Übergangslösung. : Geflüchtete aus der Ukraine erhalten Behandlungsscheine –Hinweise für Praxen [Internet]. Kassenärztliche Bundesverwaltung. 2022 [cited 2022Nov28]. Available from: https://www.kbv.de/html/1150_57290.php#:~:text=08.03.2022%20%2D%20Die%20medizinische%20Versorgung,Menschen%20einen%20Arzt%20aufsuchen%20k%C3%B6nnen.

[44] de la Hoz PF (2004). Familienleben und Gesundheit. Europäische Betrachtungen aus der Perspektive sozialer Inklusion. Diskurs.

[45] Bartsch A, Kloß A (2019). Personalized charity advertising. Can personalized prosocial messages promote empathy, attitude change, and helping intentions toward stigmatized social groups?. Int J Advert.

[46] Schubert S, Kluge U, Klapprott F, Ringeisen T. Data of: Cross-sectional survey on Germans’ awareness for refugees’ information barriers (Version 1) [Data set]. Zenodo. 2000: 10.5281/zenodo.7360136

[47] Schubert S, Kluge U, Klapprott F, Ringeisen T. Mplus code: Cross-sectional survey on Germans\‘ awareness for refugees\‘ information barriers. 2022: 10.5281/zenodo.7360341

[48] Maehler D. Akkulturation und Identifikation bei eingebürgerten Migranten in Deutschland. Münster: Waxmann Verlag, 2021

